# Insulin resistance and periodontitis: Mediation by blood pressure

**DOI:** 10.1111/jre.13333

**Published:** 2024-08-09

**Authors:** Ashish C. Kalhan, Tosha A. Kalhan, Mario Romandini, Fernando V. Bitencourt, Upul M. P. Cooray, Fábio R. M. Leite, Gustavo G. Nascimento

**Affiliations:** ^1^ National Dental Research Institute Singapore, National Dental Centre Singapore Singapore Singapore; ^2^ Saw Swee Hock School of Public Health National University of Singapore Singapore Singapore; ^3^ Department of Periodontology, Faculty of Dentistry University of Oslo Oslo Norway; ^4^ Department of Dentistry and Oral Health, Section for Oral Ecology Aarhus University Aarhus Denmark; ^5^ Steno Diabetes Center Aarhus Aarhus University Hospital Aarhus Denmark; ^6^ Oral Health Academic Clinical Programme Duke‐NUS Medical School Singapore Singapore

**Keywords:** endothelial dysfunction, hypertension, obesity, periodontal diseases, risk factors, triglycerides

## Abstract

**Aim:**

This study investigated the association between the triglyceride‐glucose (TyG) index, a surrogate marker of insulin resistance, and moderate/severe periodontitis and the role of blood pressure as a mediator in this association. A second aim was to assess the role of cardiometabolic conditions such as obesity, hypertension, and dyslipidemia as potential effect modifiers.

**Methods:**

Data from 5733 US adults aged 30–64 years and with complete periodontal examination were analyzed (NHANES 2011–2014). Participants were classified as having moderate/severe periodontitis or mild/no periodontitis according to the CDC/AAP criteria as the outcome. The exposure was the TyG index, while both systolic (SBP), and diastolic (DBP) blood pressure were tested as mediators using parametric g‐formula. Analyses were adjusted for relevant confounders, namely, age, sex, ethnicity, poverty‐income ratio, and smoking, using inverse probability treatment weighting. Obesity status (based on a body mass index ≥30 kg/m^2^), self‐report of hypertension and dyslipidemia (calculated based on the thresholds provided by National Cholesterol Education Program‐Adult Treatment Panel‐III) were tested as effect modifiers.

**Results:**

The findings showed the TyG index to be associated with increased odds of moderate/severe periodontitis [odds ratio (OR), 95% confidence interval (CI) = 1.17 (1.11–1.23)], with 50% of the total effect mediated by SBP. Stratified analysis showed a stronger association in individuals with obesity, hypertension, and dyslipidemia compared to those without these conditions. However, in those taking anti‐hypertensive medications, the association was partially mitigated. Sensitivity analysis using imputed data showed consistent results.

**Conclusion:**

The TyG index was associated with increased odds of moderate/severe periodontitis, especially in individuals with obesity, hypertension, and dyslipidemia. SBP levels partially mediated this association.

## Graphical abstract

Every unit increase in the triglyceride‐glucose index (TyG index) was associated with 17% increased odds of moderate/severe periodontitis, with a stronger association seen in individuals with obesity, hypertension, and dyslipidemia. Sensitivity analyses post‐imputation showed consistent results. Pathway analysis showed systolic blood pressure to mediate about 50% of the total association between TyG index and moderate/severe periodontitis.

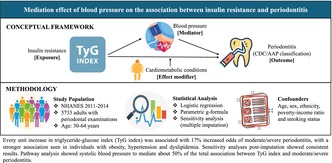

**Clinical Relevance Box**

**Background**

Although studies have shown insulin resistance to be associated with periodontitis, evidence regarding the underlying mechanisms is still unclear.

**Added Value of this Study**

This is the first study reporting potential mechanistic pathways linking triglyceride‐glucose index (TyG index), a validated surrogate marker of insulin resistance, and moderate/severe periodontitis using a large population‐representative sample. Utilizing reliable analytical approaches, such as parametric g‐formula, our findings show that systolic blood pressure partially mediates the association of TyG index and moderate/severe periodontitis, with a stronger association observed in individuals with obesity, hypertension, and dyslipidemia.

**Clinical Implications**

Our findings highlight a potential mechanistic link between cardiometabolic dysregulation and periodontal health, emphasizing the multifaceted interplay of systemic factors in the occurrence of periodontitis.

## 1 INTRODUCTION

Type 2 diabetes mellitus (T2DM) is a well‐known risk factor for periodontitis.^1^ Insulin resistance, a key player in the development of T2DM,^2^ has also been shown to be associated with periodontitis,^3^, ^4^, ^5^ even in adolescents.^6^, ^7^ Although an increase in systemic inflammation has been proposed as the most plausible mechanism,^5^ specific mechanistic pathways explaining the association remain largely unclear.

Evidence suggests that insulin resistance is associated with endothelial dysfunction, possibly through the impairment of insulin‐related signaling pathways.^8^ This dysfunction may, in turn, disrupt the balance between nitric oxide (NO)‐dependent vasodilator and endothelin‐1‐dependent vasoconstrictor actions,^9^ resulting in alterations in blood pressure.^10^ Similarly, investigations have shown both insulin resistance and blood pressure to be associated with a reduced abundance of oral nitrate‐reducing bacteria in the subgingival biofilm.^11^ However, no study has yet explored the mediating role of blood pressure in the association between insulin resistance and periodontitis.

The gold standard for assessing insulin resistance is the hyperinsulinemic–euglycemic clamp analysis.^12^ Its application in real‐world settings is, however, challenging, as it is time‐consuming; hence, the homeostatic model assessment of insulin resistance (HOMA‐IR) has been used as a comparable yet simpler and more time‐efficient method.^13^ Nevertheless, this method has limited utility in clinical settings because serum insulin is not routinely measured. Alternatively, the triglyceride glucose index (TyG index) has been proposed as an accessible and cost‐effective surrogate marker for evaluating insulin resistance,^14^ with high sensitivity and specificity.^15^

Given the above‐mentioned gaps, this study investigated the association between the TyG index and periodontitis while assessing the role of blood pressure as a potential mediator in the association. Since a higher prevalence of periodontitis has been reported in obese individuals with insulin resistance,^4^, ^16^ the second aim was to investigate whether obesity status modifies this association.

## 2 METHODS

The present manuscript is reported according to the STrengthening the Reporting of OBservational studies in Epidemiology (STROBE) guidelines.^17^

### 2.1 Study population and design: NHANES 2011–2014

This study used data from individuals participating in the 2011–2012 and 2013–2014 cycles of the National Health and Nutrition Examination Survey (NHANES). NHANES is a nationally representative population‐based cross‐sectional survey among civilians in the US and contains information from a stratified, multistage probability sample of non‐institutionalized individuals. Detailed descriptions of the survey design, interview, and examinations have been previously published.^18^, ^19^ Informed consent was obtained from all participants, and data collection protocols were approved by the NCHS Research Ethics Review Board.

**FIGURE 1:**
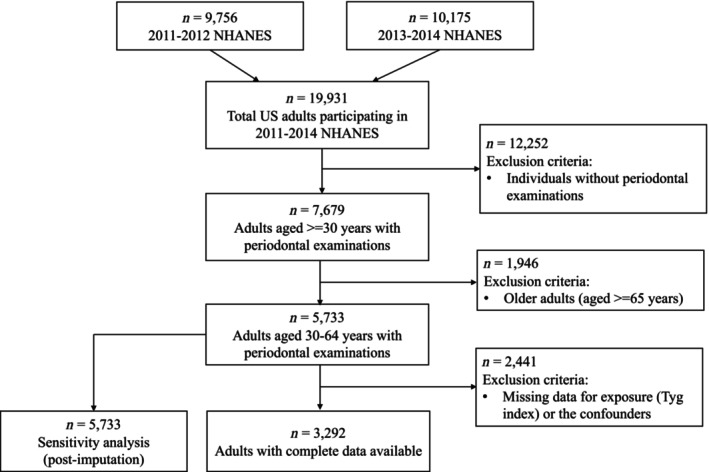
Flowchart describing the selection of the study participants.

In total, 19 931 individuals participated in the 2011–2012 and 2013–2014 NHANES cycles.^19^ Our analyses were limited to adults aged 30–64 years who completed periodontal examination (*n* = 5733) (Figure 1). The rationale for such age limitation was as follows: (a) periodontal examinations were only conducted in dentate adults ≥30 years of age, and (b) from age 65 and above, a higher proportion of the population was observed to be edentulous (22.6% vs. 3.9%), which would underestimate the true association of insulin resistance with periodontitis since it is not possible to ascertain the cause of edentulousness due to the NHANES study design.

### 2.2 Triglyceride‐glucose index (exposure)

Blood specimens were collected using standard procedures and stored appropriately until further analysis. The TyG index was calculated as ln[fasting triglycerides (mg/dL) × fasting glucose (mg/dL)/2], as previously described,^15^ and was used as a continuous variable in all analyses.

### 2.3 Periodontitis (outcome)

Full‐mouth periodontal examinations were performed by calibrated dentists using a color‐coded periodontal probe (PCP2, HuFriedy). Gingival recession and probing pocket depth (PPD) were measured at six sites per tooth (excluding third molars), and the clinical attachment level (CAL) was then calculated. For overall CAL and PPD across all periodontal sites, the inter‐class coefficients (ICCs) ranged from 0.80 to 0.90 and 0.79 to 0.86 in the 2011–2012 and 2013–2014 cycles, respectively. Further details about the periodontal examination are described elsewhere.^19^ The CDC/AAP case definitions were used to classify participants into moderate/severe periodontitis and mild/no periodontitis (reference category) groups.

### 2.4 Blood pressure (mediator)

Blood pressure (BP) measurements were taken during a single visit using a mercury wall model sphygmomanometer (Baumanometer, Baum) with standard Bauman cuffs. The averages of three brachial readings of systolic (SBP) and diastolic (DBP) values, respectively, were considered.^20^

### 2.5 Confounders and effect modifier

The considered a priori confounders [age, sex, ethnicity, income (poverty‐income ratio), and smoking status] were self‐reportedly assessed through interviews with structured questionnaires. Age was categorized into ≤47 years and >47 years (cut‐off based on the mean age of the study participants). The poverty‐income ratio was categorized as <=1.08, 1.09–2.81, and >=2.81 and was calculated by dividing the midpoint of the observed family income by the poverty threshold, the age of the family reference person, and the calendar year in which the family was interviewed. Smoking status was categorized as never, former, and current smokers.^21^

Obesity status was assessed using the body mass index (BMI), calculated as weight in kilograms divided by height in meters squared, and categorized as non‐obese (BMI <30 kg/m^2^) and obese (BMI ≥30 kg/m^2^). To classify dyslipidemia, the following cutoff values were adopted according to the National Cholesterol Education Program (NCEP) Adult Treatment Panel‐III (ATP‐III): triglycerides ≥150 mg/dL; total cholesterol >200 mg/dL; LDL >100 mg/dL, or HDL <40 mg/dL in males and 50 mg/dL in females. Participants with ≥1 lipid biomarker above the threshold were considered as having dyslipidemia. The presence of comorbidities such as history of type 2 diabetes, cardiovascular disease, hypertension, and those taking anti‐hypertensive medications were recorded based on self‐reported measures.

### 2.6 Statistical analyses

**FIGURE 2:**
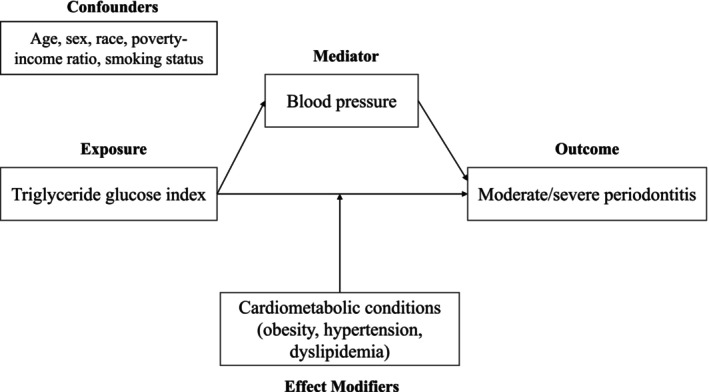
Conceptual framework used in the analysis.

Figure 2 illustrates the conceptual framework used to guide the covariates selection as well as the analytical approach employed in the study.

Descriptive statistics, stratified by the outcome, were initially used to characterize the subjects using weighted frequencies of categorical variables and the mean (± standard error, SE) and 95% confidence interval for continuous variables. Chi‐square tests were employed to assess the differences in the covariates with respect to the outcome.

Logistic regression analyses adjusted for confounders were then used to assess the association between the TyG index (exposure, continuous variable) and periodontitis (outcome, dichotomous variable). The parametric g‐formula was used to test the total effect, natural direct/indirect effects, and controlled direct effect of the TyG index (continuous variable) on periodontitis, as well as the proportion of the total effect mediated by SBP/DBP. The Monte Carlo approach was used to estimate the effects. A total of 1000 bootstrap samples were used to estimate the standard errors as well as the confidence interval of the estimated effects. Sensitivity analyses were performed employing predictive mean matching using multiple imputation by chained equation (MICE) in 10 imputed datasets to assess the potential bias and influence of missing data on the study findings.

Stata version 18 (Stata Corp., College Station) was used for data analysis, considering the appropriate survey sample weights. *p*‐values <.05 were considered statistically significant.

## 3 RESULTS

### 3.1 Study population

Table 1 shows the weighted characteristics of the study population with respect to periodontal status. The overall sample's mean age was 47 years, with a 42% prevalence of moderate/severe periodontitis. Statistically significant differences in sex, age, ethnicity, poverty‐income ratio, and smoking status were observed between moderate/severe periodontitis and mild/no periodontitis (all *p* < .05). A greater mean TyG index was observed in individuals with moderate/severe periodontitis compared to those with mild/no periodontitis (8.72 vs. 8.56; *p* = .04).

**TABLE 1:** Participant characteristics of 5733 US adults (aged 30–64 years) with and without moderate/severe periodontitis (NHANES, 2011–2014).

Variables	Moderate/severe periodontitis *n* = 2410		Mild and no periodontitis *n* = 3323		*p*‐value^b^
	*n*	% (95% CI)^a^	*n*	% (95% CI)^a^	
TyG index					<.001
>50th percentile (>8.55)	581	25.42 (22.40–28.70)	738	22.34 (20.59–24.20)	
<=50th percentile (<=8.55)	494	19.97 (17.17–23.11)	826	24.87 (22.86–26.99)	
Missing	1335	54.60 (51.08–58.08)	1759	52.79 (50.37–55.20)	
Sex					<.001
Male	1401	59.99 (57.55–62.38)	1405	43.97 (41.96–46.00)	
Female	1009	40.01 (37.62–42.45)	1918	56.03 (54.00–58.04)	
Race					<.001
Hispanics	652	20.79 (15.95–26.65)	638	11.69 (9.12–14.84)	
Non‐Hispanic Whites	674	53.77 (46.58–60.81)	1494	72.24 (67.99–76.13)	
Non‐Hispanic Blacks	697	16.44 (12.40–21.46)	619	8.53 (6.90–10.51)	
Non‐Hispanic Asians	325	6.23 (4.89–7.91)	468	5.05 (3.87–6.56)	
Others	62	2.76 (1.79–4.21)	104	2.48 (1.82–3.38)	
Poverty‐income ratio (PIR)					<.001
0–1.08	726	23.01 (19.83–26.53)	616	11.67 (9.51–14.23)	
1.09–2.81	800	32.41 (30.14–34.77)	862	22.78 (19.96–25.87)	
2.82–5.0	654	36.99 (33.58–40.53)	1634	60.47 (55.76–65.00)	
Missing	230	7.59 (5.95–9.62)	211	5.08 (4.08–6.30)	
Smoking status					<.001
Current smoker	745	33.30 (30.99–35.70)	558	15.16 (13.24–17.29)	
Former smoker	537	24.54 (22.12–27.13)	673	22.27 (19.78–24.99)	
Never smoker	1125	42.08 (39.49–44.72)	2092	62.57 (59.83–65.23)	
Missing	3	0.07 (0.02–0.24)	0	‐	
Presence of obesity^c^					.003
Obese	962	40.79 (37.28–44.39)	1190	35.28 (32.83–37.81)	
Non‐obese	1411	57.92 (54.32–61.44)	2092	63.71 (61.15–66.19)	
Missing	37	1.29 (0.84–1.96)	41	1.01 (0.64–1.57)	
Presence of diabetes^d^					<.001
Yes	350	12.04 (10.78–13.42)	274	6.84 (5.73–8.16)	
No	1983	84.95 (83.40–86.38)	2975	91.06 (89.50–92.41)	
Missing	77	3.00 (2.17–4.15)	74	2.09 (1.62–2.69)	
Presence of CVD^e^					<.001
Yes	169	6.61 (5.42–8.05)	139	3.32 (2.66–4.12)	
No	2227	93.06 (91.67–94.24)	3183	96.66 (95.85–97.31)	
Missing	14	0.31 (0.17–0.57)	1	0.02 (0.002–0.16)	
Presence of hypertension^f^					<.001
Yes	909	36.77 (33.96–39.66)	972	28.07 (25.78–30.48)	
No	1499	63.20 (60.32–65.99)	2350	71.91 (69.50–74.20)	
Missing	2	0.03 (0.007–0.13)	1	0.02 (0.002–0.15)	
Prescribed anti‐hypertensive medications					<.001
Yes	766	30.90 (28.15–33.78)	762	21.81 (19.90–23.84)	
No	143	5.87 (4.76–7.21)	209	6.19 (4.95–7.71)	
Missing	1501	63.23 (60.34–66.04)	2352	72.00 (69.61–74.27)	
Presence of dyslipidemia^g^					.019
Yes	1728	72.75 (69.51–75.77)	2301	70.52 (68.07–72.86)	
No	183	7.24 (5.78–9.04)	320	9.25 (8.12–10.51)	
Missing	499	20.00 (17.92–22.25)	702	20.23 (18.21–22.40)	
Prescribed cholesterol lowering medications					<.001
Yes	575	23.59 (21.45–25.88)	630	19.57 (17.68–21.62)	
No	1190	50.49 (47.38–53.60)	2037	63.23 (61.07–65.35)	
Missing	645	25.91 (23.11–28.93)	656	17.19 (15.46–19.07)	

	*n*	Mean (SE)	*n*	Mean (SE)	*p*‐value^c^
TyG index	1075	8.73 (0.04)	1564	8.58 (0.02)	<.001
Age (in years)	2410	48.87 (0.34)	3323	45.52 (0.29)	<.001
HbA1c (%)	2320	5.85 (0.03)	3210	5.54 (0.02)	<.001
Fasting plasma glucose (mg/dl)	1089	112.54 (1.40)	1578	102.70 (0.65)	<.001
Triglycerides (mg/dl)	1079	141.94 (7.10)	1565	130.16 (4.22)	.003
Systolic blood pressure (mm Hg)	2153	123.84 (0.59)	3004	118.33 (0.39)	<.001
Diastolic blood pressure (mm Hg)	2153	73.43 (0.35)	3004	72.38 (0.37)	.0002

Abbreviations: CI, confidence interval; CVD, cardiovascular diseases; HbA1c, glycated hemoglobin; TyG index, triglyceride glucose index.

^a^ Weighted percent and 95%CI provided by NCHS.

^b^ *p*‐values are based on the Chi‐square test.

^c^ Presence of obesity was classified based on the BMI levels: Obese = BMI≥30 mg/kg^2^ and non‐obese = BMI < 30 mg/kg^2^.

^d^ Presence of diabetes was defined as a self‐report of ever being told by a doctor to have diabetes.

^e^ Presence of CVD was defined as a self‐report of either of the following: congestive heart failure, coronary heart disease, angina/angina pectoris, stroke, or heart attack.

^f^ Presence of hypertension was defined as a self‐report of ever being told by a doctor of having high blood pressure.

^g^ To classify dyslipidemia, the following cutoff values were adopted according to the National Cholesterol Education Programme (NCEP) Adult Treatment Panel‐III (ATP‐III): triglycerides ≥150 mg/dL; total cholesterol >200 mg/dL; LDL >100 mg/dL, or HDL <40 mg/dL in males and 50 mg/dL in females. Participants with ≥1 lipid biomarker above the threshold were considered as having dyslipidemia.

### 3.2 Association between TyG index and periodontitis

After adjusting for potential confounders, the multivariable analysis demonstrated the TyG index to be associated with increased odds of moderate/severe periodontitis (OR = 1.17, 95% CI, 1.00–1.36) (Table 2). Stratified analysis showed a stronger association in individuals with cardiometabolic comorbidities such as obesity [obesity: (OR = 1.31, 95% CI 1.06–1.60; no‐obesity: OR = 1.06, 95% CI 0.79–1.41]), hypertension [hypertensive: OR = 1.22, 95% CI 1.01–1.47; no‐hypertension: OR = 1.09, 95% CI 0.85–1.40], and dyslipidemia [dyslipidemia: OR = 1.18, 95% CI 1.01–1.37; no‐dyslipidemia: OR = 0.83, 95% CI 0.42–1.64]. However, in individuals taking anti‐hypertensive medications, the association was partially mitigated. Sensitivity analyses post‐imputation showed consistent results (Table 2).

**TABLE 2:** Multivariable analysis investigating TyG index‐moderate/severe periodontitis association and assessing the role of cardiometabolic comorbidities as effect modifiers.

	Outcome: Moderate/severe periodontitis									
	All individuals		Stratified by comorbidities^d^							
			Individuals with obesity^a^		Individuals with hypertension^b^		Individuals with hypertension^b^ and prescribed anti‐hypertensive medications		Individuals with dyslipidemia^c^	
	*N*	Adjusted OR (95% CI)	*N*	Adjusted OR (95% CI)	*N*	Adjusted OR (95% CI)	*N*	Adjusted OR (95% CI)	*N*	Adjusted OR (95% CI)
Complete case analysis										
TyG index	3292	1.17 (1.00–1.36)*	1072	1.31 (1.06–1.60)*	882	1.22 (1.01–1.47)*	723	1.16 (0.93–1.45)	2134	1.18 (1.01–1.37)*
Post‐imputation**										
TyG index	5733	1.10 (0.95–1.29)	2189	1.13 (0.90–1.42)	1881	1.11 (0.91–1.37)	1528	1.05 (0.78–1.40)	4029	1.12 (0.92–1.35)

*Note*: Adjusted for age, sex, race, poverty‐income ratio, and smoking status.

Abbreviations: CI, confidence interval; OR, odds ratio; TyG index, triglyceride‐glucose index.

^a^ Presence of obesity was classified based on the BMI levels: Obese = BMI≥30 mg/kg^2^ and non‐obese = BMI < 30 mg/kg^2^.

^b^ Presence of hypertension was defined as a self‐report of ever being told by a doctor of having high blood pressure.

^c^ To classify dyslipidemia, the following cutoff values were adopted according to the National Cholesterol Education Programme (NCEP) Adult Treatment Panel‐III (ATP‐III): triglycerides ≥150 mg/dL; total cholesterol >200 mg/dL; LDL >100 mg/dL, or HDL <40 mg/dL in males and 50 mg/dL in females. Participants with ≥1 lipid biomarker above the threshold were considered as having dyslipidemia.

^d^ Stratification by Type 2 diabetes could not be performed owing to the limited number of diabetic individuals in the study.

*p ≤ .05.

**Predictive mean matching using multiple imputations by chained equation (MICE) in 10 imputed datasets.

### 3.3 Mediation by blood pressure

For systolic blood pressure (SBP), the total effect (*β* = .16, 95% CI 0.10–0.22) of the TyG index on moderate/severe periodontitis, as well as the natural indirect effect (*β* = .08; 95% CI 0.04–0.12) and the natural direct effect (*β* = .08; 95% CI 0.01–0.14), were statistically significant, with 50% of the total effect mediated by SBP (Table 3). The controlled direct effect of the TyG index on moderate/severe periodontitis at SBP = 121 mm Hg was *β* = .11 (95% CI 0.04–0.19). In contrast, a weaker mediation effect of diastolic blood pressure (DBP) on the Tyg index‐moderate/severe periodontitis association was observed. Sensitivity analyses post‐imputation showed consistent results. Additional analysis in individuals taking anti‐hypertensive medications showed no mediation effect of blood pressure (SBP or DBP).

**TABLE 3:** G‐computation analysis to assess the role of blood pressure as a mediator in the association of TyG index and moderate/severe periodontitis.

		Outcome: Moderate/severe periodontitis				
		CDE^b^	NDE	NIE	TCE	PM^a^
Exposure	Mediator	Estimate (95% CI)	Estimate (95% CI)	Estimate (95% CI)	Estimate (95% CI)	Estimate (95% CI)
Complete case analysis (*n* = 3890)						
TyG index	SBP	0.11 (0.04–0.19)*	0.08 (0.01–0.14)*	0.08 (0.04–0.12)*	0.16 (0.10–0.22)*	0.50 (0.25–0.76)*
	DBP	0.14 (0.07–0.20)*	0.10 (0.04–0.17)*	0.04 (−0.001–0.09)	0.15 (0.09–0.21)*	0.29 (0.02–0.56)*
Post‐imputation (*n* = 5733)						
TyG index	SBP	0.07 (0.01–0.13)*	0.12 (0.06–0.18)*	0.07 (0.03–0.11)*	0.19 (0.13–0.25)*	0.37 (0.06–067)*
	DBP	0.10 (0.04–0.15)*	0.15 (0.09–0.21)*	0.04 (−0.0090.08)	0.19 (0.13–0.25)*	0.18 (−0.190–0.57)
Sensitivity analysis [Individuals prescribed anti‐hypertensive medications (*n* = 1528)]						
TyG index	SBP	0.06 (−0.04–0.16)	0.08 (−0.01–0.19)	0.02 (−0.03–0.06)	0.10 (0.00–0.20)*	0.15 (−2.94–3.23)
	DBP	0.06 (−0.04–0.16)	0.09 (−0.008–0.20)	0.003 (−0.04–0.05)	0.10 (−0.005–0.20)*	0.03 (−2.58–2.64)

*Note*: Adjusted for age, sex, race, poverty‐income ratio, smoking status, presence of obesity, hypertension, and dyslipidemia. Point estimates and 95% CI were estimated by parametric g‐computation in 1000 bootstrapped datasets.

Abbreviations: CDE, controlled direct effect; CI, confidence interval; DBP, diastolic blood pressure; NDE, natural direct effect; NIE, natural indirect effect; PM, proportion mediated; SBP, systolic blood pressure; TCE, total causal effect; Tyg index, triglyceride‐glucose index.

^a^ Proportion mediated (PM) is calculated as estimates of NIE/TCE.

^b^ CDE estimated when the mediator is set at the following mean value: SBP = 121 mm Hg; DBP = 72 mm Hg.

*p ≤ .05.

## 4 DISCUSSION

To the best of the authors' knowledge, this is the first study reporting potential mechanistic pathways linking insulin resistance and periodontitis using a large population‐representative sample. Our study has three main findings. First, the TyG index, a surrogate marker of insulin resistance, was associated with increased odds of moderate/severe periodontitis. Similar findings have been reported recently in American^22^ and Korean adults^23^ using periodontitis as the outcome. Second, the presence of comorbidities such as obesity, hypertension, and dyslipidemia appeared to modify this association, with the relationship more pronounced in individuals having these conditions. Third, systolic blood pressure partially mediated (50%) the association.

The relationship between insulin resistance and periodontitis unfolds through intricate molecular and cellular alterations, encompassing potential epigenetic modifications intensified by tissue hypoxia. It is suggested that the reduction in nitric oxide availability resulting from insulin resistance may trigger systemic inflammation, leading to the release of pro‐inflammatory mediators,^24^ and an increase in reactive oxygen species production, potentially caused by impaired mitochondrial function and viability resulting from an influx of fatty acids to compensate for a deficit in insulin.^25^ All these cascades of events might, in turn, also contribute to dysbiosis in the oral biofilm composition, favoring the proliferation of periodonto‐pathogenic species and diminishing bacterial species responsible for nitrate reduction to nitric oxide, an antimicrobial free radical able to inhibit anaerobes growth.^26^, ^27^

Furthermore, diminished insulin sensitivity potentially contributes to endothelial dysfunction within capillaries. This impairment may impede vasodilation and perturb the blood flow regulation.^28^ It may also exacerbate platelet aggregation and adhesion, which can contribute to small thrombus formation and inflammation, especially in the peripheral circulation.^29^ Consequently, compromised tissue oxygenation and the inadequate clearance of cytotoxic metabolites may not only induce alterations in the oral biofilm and immune responses but also exert a profound influence on resident cellular elements.^30^ For instance, the hypoxia‐inducible factor pathway, activated in hypoxic conditions, orchestrates angiogenesis, glycolysis, and inflammatory responses in periodontal cells.^31^, ^32^ Simultaneously, oxidative stress associated with hypoxia activates signaling pathways like nuclear factor‐kappa B (NF‐κB) and mitogen‐activated protein kinases (MAPK), amplifying inflammation within periodontitis lesions.^33^, ^34^

Evidence from recent epidemiological studies corroborates a potential role of circulatory dysfunction in the triglyceride and insulin relationship with oral tissues. For example, while a dose–response relationship between the TyG index and increased systolic blood pressure has been reported,^35^ insulin resistance has been associated with increased odds of developing incident prehypertension and hypertension,^36^, ^37^ suggesting a possible temporal relationship with insulin resistance preceding the development of pre‐hypertension. Possible mechanisms speculated for the development of hypertension in insulin‐resistant individuals are via (a) endothelial dysfunction resulting from pathway‐specific impairment in PI3K‐dependent signaling^28^ and/or, (b) activation of the renin‐angiotensin‐aldosterone system with compensatory hyperinsulinemia in insulin‐resistant conditions.^38^ Furthermore, hypertension has been associated with increased odds of moderate/severe periodontitis in a recent meta‐analysis.^39^

One may wonder why the effect of systolic blood pressure as a mediator was stronger than diastolic blood pressure. Inflammatory diseases showcase a complex relationship with alterations in blood pressure dynamics, particularly involving systolic blood pressure dominance due to its interconnectedness with capillary dysfunction.^40^ As such, inflammatory‐mediated vascular alterations, including remodeling and narrowing of arteries and capillaries, resulting in increased peripheral vascular resistance through vasoconstriction, have been suggested to play a predominant role in elevating systolic blood pressure while exerting less influence on diastolic pressure.^40^

In this study, obesity status appeared to modify the relationship between insulin resistance and periodontitis, with a higher TyG index associated with increased odds of moderate/severe periodontitis among obese participants. Obesity has been suggested as a contributing factor for T2DM through the stimulation of insulin resistance^41^ and has also been associated with endothelial dysfunction and hypertension. While studies have demonstrated a weaker association between the TyG index and cardiovascular outcomes in non‐obese American adults,^42^ a greater prevalence of severe periodontitis has been observed in obese individuals with insulin resistance.^4^, ^16^ Considering that insulin resistance and endothelial dysfunction are more pronounced in obese individuals,^43^ greater odds of severe periodontitis are expected in those individuals compared to non‐obese ones.

The association between TyG index and moderate/severe periodontitis was consistent in individuals with a self‐report of high blood pressure. However, the limited sample size did not allow further comparisons of the strength of IR‐periodontitis association between those taking antihypertensive medications and those who did not. Nevertheless, subgroup analysis in those prescribed anti‐hypertensive medications showed reduced estimates (~50% reduction) compared to the overall estimates in individuals with hypertension (Table 2). A cautious interpretation of these results is warranted owing to the observational nature and cross‐sectional design of the study. Future randomized trials could explore the effect of controlling blood pressure in individuals with high insulin resistance on the risk of periodontal disease.

Based on the TyG threshold for insulin resistance reported previously in the Mexican population (TyG >4.68),^14^, ^15^ all individuals in the analyzed US population were estimated to have some form of insulin resistance, with similar mean TyG values reported in other studies using NHANES data.^44^ To further estimate the prevalence of moderate/severe periodontitis based on the severity of IR, we used the 50th percentile of the observed TyG values as the threshold to classify those with greater and lesser IR severity. Our findings showed the prevalence of moderate/severe periodontitis to be greater in individuals with more severe IR (higher TyG; 44.04%) compared to those with less severe IR (lower TyG; 37.44%). Conversely, it is also true that ~56% of individuals with higher TyG index had mild or no periodontitis, which points to the complex interplay of various pathogenic mechanisms involved, in addition to IR severity, leading to the development of periodontal disease. For instance, the prevalence of hypertension was greater in those with a higher TyG index and moderate/severe periodontitis (44.4%) compared to those with a higher TyG index and mild/no periodontitis (38.5%). Lastly, in such observational studies, the issue of residual confounding also needs to be considered while interpreting the study findings.

Given the cross‐sectional design of the NHANES survey, a cautious interpretation of the cause‐and‐effect relationship is warranted. Although biological evidence from prospective longitudinal studies supports the rationale behind the proposed hypothesis, one cannot rule out the possibility of reverse causation. Since insulin resistance and hypertension are closely linked and often coexist, the mediating effect of IR in the association between BP and periodontitis was also explored; however, no mediation effect was observed. Evidence from the literature suggests while diabetes, a predominant consequence of IR, may causally affect hypertension, the inverse causal link appears to be weak.^45^ Further longitudinal studies are warranted to understand the intricate relationship between insulin resistance, hypertension, and periodontitis. Another limitation relates to the absence of data on bleeding on probing, a relevant parameter for describing periodontal inflammation. While a non‐linear relationship between TyG index and periodontitis has been explored in American adults, the results indicated a linear relationship after the cutoff of approximately 8.5 (median value) was modeled,^22^ thus corroborating our methodological choice. The strength of the study lies in three key points. First, it used a nationally representative sample of US adults, ensuring wide demographic coverage and statistical power. Second, the use of data from a complete periodontal examination protocol minimized the risk of information bias. Third, it utilized analytical approaches, such as the parametric g‐formula and inverse probability treatment weighting, which enable more reliable inferences while controlling for confounding variables in a more balanced use of covariates across different groups.

## 5 CONCLUSIONS

The triglyceride‐glucose index was associated with increased odds of moderate/severe periodontitis, especially in individuals with cardiometabolic conditions such as obesity, hypertension, and dyslipidemia. Mediation analysis showed the association to be partially explained by systolic blood pressure, highlighting a potential mechanistic link between cardiometabolic dysregulation and periodontal health and emphasizing the multifaceted interplay of systemic factors in the occurrence of periodontitis.

## CONFLICT OF INTEREST STATEMENT

No conflict of interest to disclose.

## Data Availability

The data that support the findings of this study are openly available in NHANES at www.cdc.gov/nchs/nhis/index.htm.
